# Pediatric human immunodeficiency virus infection in the modern era: A systematic review of recent advances and their implications

**DOI:** 10.1002/ped4.70019

**Published:** 2025-08-25

**Authors:** Klaudia Nowak, Krzysztof Łupina, Dominika Lorek, Aleksandra Jabłońska, Alicja Radomska, Tymon Choromański, Jakub Janczura

**Affiliations:** ^1^ Collegium Medicum Jan Kochanowski University Kielce Poland

**Keywords:** Antiretroviral therapy, Children, Human immunodeficiency virus, Long‐acting injectables, Pediatric

## Abstract

**Importance:**

Pediatric human immunodeficiency virus (HIV) infection continues to pose a significant global health burden, especially in low‐ and middle‐income countries. Despite advances in antiretroviral therapy (ART), children living with HIV face unique clinical, developmental, and systemic challenges.

**Objective:**

To systematically review recent developments in pediatric HIV care, with a focus on treatment innovations, complications, and the transition from pediatric to adult care.

**Methods:**

A comprehensive literature review was conducted across PubMed, Scopus, EMBASE, and the World Health Organization databases, covering studies published between 2012 and 2025. Inclusion criteria focused on original research, clinical trials, and guidelines addressing pediatric ART, long‐acting therapies, prevention strategies, treatment complications, and transitional care.

**Results:**

Early ART initiation was associated with improved neurodevelopment and reduced disease progression. Current pediatric ART regimens favor simplified combinations and weight‐based dosing, but pharmacokinetic variability, toxicity, and adherence remain concerns. Long‐acting injectable therapies, such as cabotegravir/rilpivirine and investigational agents like lenacapavir and islatravir, show promise for adolescents. Prevention of mother‐to‐child transmission has significantly reduced pediatric HIV incidence, largely through maternal ART and pre‐exposure prophylaxis. However, stigma, poor awareness, and healthcare disparities hinder broader impact. Children on lifelong ART face increased risks of metabolic, cardiovascular, renal, and neurocognitive complications. Transitioning to adult care remains a vulnerable period with high rates of treatment disengagement.

**Interpretation:**

Advances in pediatric HIV care are substantial but uneven. Continued investment in age‐appropriate therapies, psychosocial support, and implementation research is essential to close persistent gaps and ensure equitable, lifelong care for children and adolescents living with HIV.

## Graphical abstract

This review discusses how early antiretroviral therapy initiation, long‐acting therapies, and prevention of mother‐to‐child transmission contribute to improved pediatric human immunodeficiency virus outcomes. Despite these advances, treatment complications and challenges in transitioning to adult care remain major barriers to lifelong management.

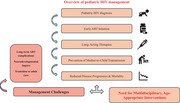

## INTRODUCTION

Human immunodeficiency virus (HIV) infection remains a significant global health challenge, with pediatric populations representing a unique and vulnerable subgroup. According to the World Health Organization (WHO), an estimated 1.4 million children aged 0–14 were living with HIV at the end of 2023. That same year, approximately 120 000 new pediatric HIV infections occurred, and 76 000 children died from acquired immune deficiency syndrome (AIDS)‐related causes.^1^ Although significant progress has been achieved in the prevention, diagnosis, and treatment of pediatric HIV, the burden of disease remains substantial, particularly in low‐ and middle‐income countries. The highest numbers and prevalence of HIV‐positive individuals are concentrated in regions such as Latin America, Eastern Europe, and East/Southeast Asia, underscoring the ongoing challenges in HIV control within resource‐limited settings.^2^ Pediatric HIV is characterized not only by unique virological and immunological dynamics but also by a range of clinical, developmental, and psychosocial complications that differ from those observed in adult populations.^3^ Infants and young children are especially susceptible to rapid disease progression due to their immature immune systems and heightened viral replication rates.^3^ Over the past decades, the introduction and evolution of antiretroviral therapy (ART) have transformed the prognosis of pediatric HIV, significantly reducing morbidity and mortality.^4^ However, optimizing ART in children involves complex considerations, including age‐appropriate formulations, pharmacokinetics, safety profiles, and adherence challenges. In recent years, novel therapeutic strategies such as long‐acting injectables (LAIs) and refined ART combinations have emerged, aiming to simplify treatment and improve outcomes.^4^ This review provides a comprehensive analysis of recent developments in pediatric HIV management, emphasizing their clinical implications and identifying areas for further research and innovation.

## METHODS

A comprehensive literature review was conducted using PubMed, Scopus, EMBASE, and Web of Science databases, as well as official repositories from the WHO. The search strategy incorporated a combination of MeSH terms and keywords, including: (“HIV in children” OR “pediatric HIV” OR “vertical transmission” OR “antiretroviral therapy in children” OR “long‐acting HIV treatment” OR “HIV neurodevelopment” OR “HIV vaccine trials”) AND (“prevention” OR “treatment outcomes” OR “drug resistance” OR “transition to adult care” OR “clinical trials” OR “ART complications”). The search was restricted to publications between January 2012 and February 2025 to ensure up‐to‐date relevance. The review included peer‐reviewed original research articles, clinical trials, systematic reviews, and policy reports published in English. National guidelines and global HIV policy documents from the WHO and Centers for Disease Control and Prevention were also included to provide the current clinical context. Abstracts and studies were included if they addressed one or more of the following: (1) developments in pediatric ART regimens; (2) innovations such as long‐LAIs; (3) outcomes of mother‐to‐child transmission (MTCT) strategies; (4) HIV‐related complications in children; (5) neurodevelopmental outcomes; and (6) the transition of adolescents from pediatric to adult HIV care. Exclusion criteria comprised animal or in vitro studies without human data, non‐peer‐reviewed commentaries, studies lacking clinical outcome data, and articles unrelated to pediatric or adolescent populations. In total, 471 publications and reports were screened. Finally, 63 full‐text articles and reports were included in this review, with particular emphasis placed on those published after 2020 to reflect the most recent evidence. Data was extracted and synthesized thematically according to major developments in pediatric HIV diagnosis, treatment, prevention, and care.

## RESULTS

### HIV life cycle

The HIV‐1 replication cycle consists of viral entry, reverse transcription, integration into the host genome, and production of new virions.^5^ While these stages are consistent across age groups, the dynamics differ significantly in pediatric patients.^6^ In neonates and young children, HIV‐1 replicates more efficiently due to the higher expression of transcriptional activators, cytokines, and signal transduction pathways in their immune cells.^5^ This results in elevated viral loads and more rapid progression to AIDS compared to adults. Despite these differences in replication kinetics, the fundamental steps of the viral life cycle, such as binding via gp120 to CD4 receptors, fusion mediated by gp41, reverse transcription by viral polymerase, and integration via integrase, remain unchanged.^5^

### Evolution of ART

The first antiretroviral drug approved for adults, zidovudine, was later tested in children, showing cognitive and immunological improvement in those with HIV‐associated encephalopathy.^7^ However, its use was limited by dose‐dependent neutropenia and anemia. In 1991, zidovudine was found to be safe in children at a dose of 180 mg/m^2^ every six hours.^7^ Administered to HIV‐positive pregnant women and their newborns, zidovudine reduced the risk of MTCT by 67.5% (*P* < 0.05).^7^ In 1993, its use was restricted to symptomatic children with marked immunodeficiency. The introduction of protease inhibitors (PIs), starting with saquinavir in 1995, marked a turning point, offering superior viral suppression and reduced mortality.^7^ The AIDS Clinical Trials Group 152 study showed that didanosine monotherapy had lower toxicity than combination therapy with zidovudine, and zidovudine alone was no longer considered sufficient.^8^ By 1997, triple‐drug regimens, typically two nucleoside/nucleotide reverse transcriptase inhibitors (NRTIs) plus a PI, became the standard, significantly lowering the incidence of HIV‐associated encephalopathy.^8^ Subsequent guidelines, including those from 2013 onward, progressively recommended universal ART for all HIV‐infected children.^8^ Current recommendations emphasize early treatment initiation to prevent resistance, reduce the viral reservoir, limit inflammation, and improve long‐term outcomes in perinatal infection.^9^

### Innovations in pediatric ART

**FIGURE 1:**
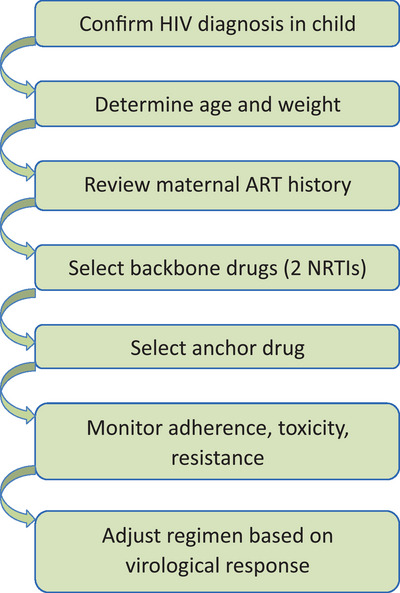
Simplified selection of antiretroviral therapy in pediatric patients. ART, antiretroviral therapy; HIV, human immunodeficiency virus; NRTIs, nucleoside reverse transcriptase inhibitors.

First‐line ART refers to the initial therapy used in the treatment of HIV‐infected individuals who have not previously been treated, to prevent replication of the virus, maintain immune system function, and improve a patient's quality of life. It is based on the administration of antiretroviral drugs from different groups.^10^ Current pediatric first‐line ART regimens consist of two NRTI backbone drugs, such as zidovudine, abacavir, or tenofovir alafenamide, combined with lamivudine, and one anchor drug from either the PI class, integrase strand transfer inhibitors, or non‐NRTIs (NNRTIs).^10^, ^11^ Simplified workflow for selecting ART in pediatric patients is presented in Figure 1. Reports on first‐line ART outcomes in children from different regions and global HIV/AIDS organizations have been extensively analyzed. Studies conducted in the UK and Ireland indicated that NNRTI‐based treatment in children resulted in virological failure.^12^ Similar findings were observed in Thailand, where initiation of NNRTI‐based regimens was linked to a higher risk of regimen changes, although a direct causal relationship with virological failure could not be confirmed.^3^ It has also been suggested that PI‐based first‐line ART is preferable for adolescents. Meanwhile, studies in Uganda supported the WHO recommendations advocating for PI‐based regimens in children younger than three years of age and highlighted the necessity of PI use in children whose mothers had received ART during breastfeeding or whose maternal treatment history was unknown.^3^, ^13^ WHO currently recommends NRTI therapy in combination with either dolutegravir or efavirenz for children weighing at least 3 kg and older than four weeks.^10^ A comparative study by Turkova et al. evaluated the efficacy of dolutegravir‐based first‐line ART, efavirenz‐based first‐line ART, and PI‐based second‐line ART boosted with ritonavir.^14^ The findings demonstrated that dolutegravir was associated with a 40% lower risk of treatment failure, provided protection against drug resistance, and ensured robust immune recovery. Furthermore, dolutegravir discontinuations were less frequent due to its favorable tolerability profile. The small tablet size and ease of administration further support its use in pediatric patients.^14^ A similar issue was observed in children, where efavirenz accelerated abacavir clearance by 12%, while the co‐administration of rifampicin and super‐boosted lopinavir reduced abacavir absorption by 29.4%.^15^ Studies conducted in Guangxi, China, assessed mortality differences among children treated with various first‐line ART regimens. The highest mortality rate (10.8%) was observed in children receiving nevirapine, whereas the use of lopinavir was associated with improved survival (3.1%). No significant difference in mortality was noted.^16^ Research on the efficacy and optimization of pediatric ART regimens is ongoing. For instance, studies investigating bictegravir/lenacapavir in children across three different age‐weight groups are currently in the recruitment phase at 15 different sites worldwide.^17^ Although numerous antiretroviral combinations exist, their use in first‐line ART is influenced by multiple factors, including drug toxicity, regional availability, financial constraints, and the age and weight of the child. Several studies in recent years have explored optimal dosing strategies. For example, Moore et al. proposed dose adjustments for dolutegravir in a study including pediatric patients from Europe, South Africa, Thailand, Uganda, and Zimbabwe.^18^ The goal was to establish a simplified and standardized dosing regimen across different weight categories. Other research efforts have focused on determining appropriate dolutegravir/lamivudine dosing,^19^ with some studies employing mathematical modeling based on real patient data to simulate optimal abacavir/dolutegravir/ lamivudine dosages.^20^, ^21^ Similarly, research has proposed fostemsavir dosing for children weighing 20 kg or more.^22^ Recent studies on weight‐based dosing in children are presented in Table 1. Despite progress in adult ART research, pediatric studies face significant challenges. Conducting clinical trials in children is complex due to multiple factors affecting drug efficacy and safety. These trials often involve numerous stages, leading to prolonged delays. One major limitation is the unavailability of pediatric formulations, making it difficult to establish bioequivalence between different dosage forms. Additionally, recruitment is hindered by the declining number of infants born with HIV, largely due to the widespread use of maternal ART. As a result, assembling a sufficient cohort of infants and young children for clinical trials remains a challenge.^23^ Other barriers include the need for personalized dosing in children with co‐infections requiring multiple antiretrovirals and the variability in pharmacokinetics across different pediatric age groups. In many cases, a single standardized dose may not be suitable for all children within the same weight or age category.^19^ Furthermore, pediatric ART trials often have small sample sizes and extended study durations, leading to multiple substudies rather than a single comprehensive trial.^23^, ^24^ Adolescents are frequently grouped with adults in clinical studies, potentially distorting the applicability of findings to pediatric populations. Another challenge in pediatric HIV treatment is accounting for rapid weight changes and organ maturation in newborns, which complicates drug dosing and delays ART initiation. Additionally, the limited availability of neonatal HIV drugs restricts treatment options. Parental concerns about experimental therapies further contribute to recruitment difficulties in pediatric ART studies.^24^ These challenges, alongside other pharmacokinetic complexities, highlight the need for continued research to optimize ART for children living with HIV.

**TABLE 1:** Weight‐based dosing according to recent studies

Study	Participants (Age/weight)	*n*	Drug	Weight (kg)	Dose
Moore et al.^18^	2–18 years ≥ 14 kg	707	Dolutegravir	14–<20 20–<25 25–<30 30–<35 35–<40 ≥ 40	20–25 mg FCT or 25 mg DT 25–50 mg FCT or 30 mg DT 25–50 mg FCT 35–50 mg FCT 35–50 mg FCT 50 mg FCT
Turkova et al.^19^	2–15 years ≥ 6 kg	386	Dolutegravir/ Lamivudine	6–<10 10–<14 14–<20 20–<25 ≥25	1.5–2.5 mg/kg DT 1.4–2.0 mg/kg DT 1.3–1.8 mg/kg DT 1.2–1.5 mg/kg DT or 2.0–2.5 mg/kg FCT ≤ 2.0 mg/kg FCT
Chandasana et al. ^20^	2 months−19 years 3.9–91.0 kg	1000	Abacavir/ Dolutegravir/ Lamivudine	6–<10 10–<14 14–<20 20–<25 25–<40	180/15/90 mg DT 240/20/120 mg DT 300/25/150 mg DT 360/30/180 mg DT 600/50/300 mg Tablet
Chandasana et al.^21^	2 months–19 years 3.9–91.0 kg	390	Abacavir/ Dolutegravir/ Lamivudine	10–<14 14–<20 20–<25 25–<40	240/20/120 mg DT 300/25/150 mg DT 360/30/180 mg DT 600/50/300 mg Tablet
Thakkar et al.^22^	18–50 years (mean age around 32 years)	32 in part 1 16 in part 2	Fostemsavir	20–<35 ≥ 35	400 mg twice daily 600 mg twice daily

Abbreviations: DT, dispersible tablet; FCT, film‐coated tablet.

### LAI therapies: a new era in HIV treatment

LAI therapies represent a promising strategy for improving adherence and viral suppression, especially in adolescents. While one LAI regimen is currently approved for pediatric use, several other agents remain under clinical investigation.

### Approved LAI therapies in pediatric HIV

LAI therapies for HIV have emerged as a promising alternative to daily oral antiretroviral regimens, offering significant advantages in terms of adherence and efficacy, particularly in pediatric populations where maintaining daily medication schedules can be challenging.^2^, ^25^ In March 2022, the Food and Drug Administration approved cabenuva—a combination of cabotegravir and rilpivirine—for use in children and adolescents aged 12 years and older who weigh at least 35 kg. Before initiating the LAI regimen, an oral lead‐in period with rilpivirine and cabotegravir may be considered to assess tolerability. Once established, the injectable formulation can be administered either monthly or every 2 months.^26^

### Investigational LAI therapies in pediatric populations

The efficacy and safety of lenacapavir in children and adolescents have not yet been established.^26^ However, findings from the PURPOSE‐2 trial demonstrated that the incidence of HIV infection was significantly lower in adults treated with lenacapavir compared to the general population.^27^ Further analysis by Ogbuagu et al. indicated that in cases of treatment interruption, oral bridging with lenacapavir (300 mg once weekly) remained effective in maintaining viral suppression.^28^ Currently, an ongoing trial is investigating the use of bictegravir/lenacapavir in children and adolescents with HIV.^29^ Additionally, a case report of a pregnant woman suggests that lenacapavir injections, with an oral lead‐in alongside other medications, could be a viable strategy for preventing MTCT while ensuring maternal and neonatal safety.^30^ Islatravir is another novel agent in Phase 3 clinical development for HIV treatment. It is being evaluated in two formulations: as part of a fixed‐dose combination with doravirine and as a stand‐alone agent.^26^ Islatravir also shows potential for long‐acting pre‐exposure prophylaxis (PrEP), particularly in an implant formulation.^31^ Clinical trials have demonstrated that treatment regimens incorporating islatravir and doravirine successfully maintained viral suppression over 96 weeks and were generally well tolerated by adult patients, regardless of the dosage level.^32^ These developments highlight the growing potential of long‐acting therapies to simplify HIV treatment and prevention, especially in populations where adherence is a challenge. Ongoing pediatric trials will be crucial in determining their safety, efficacy, and broader applicability in younger age groups. Currently used LAI and their specificities are listed in Table 2.

**TABLE 2:** Long‐acting injectable types and their use in clinical practice

Substances	Type	Used in clinical practice?
Cabotegravir+Rilpivirine	INSTI + NNRTI	Yes
Lenacapavir	Capsid inhibitor	Yes
Islatravir	NRTTI	No

Abbreviations: INSTI, integrase strand transfer inhibitor; NNRTI, non‐nucleoside reverse transcriptase inhibitor; NRTTI, nucleoside reverse transcriptase translocation inhibitor.

### Advances in the prevention of pediatric HIV

Preventing MTCT of HIV remains a critical component of global HIV control strategies, with significant progress made over the past decades due to expanded access to ARTs and preventive interventions. A substantial decline in MTCT of HIV in pediatric populations has been largely attributed to two main factors: a decrease in new HIV infections among women and expanded access to ART for pregnant and breastfeeding women. Despite these gains, the rate of decline has slowed in recent years. In 2023, an estimated 84% of pregnant women living with HIV received ART and related prevention services, up significantly from 49% in 2010.^33^ Regional studies have confirmed the positive impact of prevention of MTCT programs. For instance, a longitudinal study conducted in South‐Central China from 2010 to 2016 showed a reduction in MTCT rates from 19.4% to 9.6%, correlated with increased premarital care, HIV testing coverage among pregnant women, and drug interventions for HIV‐positive mothers and their infants.^34^ Similarly, a study in Nantong City (2012–2018) found zero cases of HIV transmission among 24 children whose mothers received ART during pregnancy.^35^ WHO's 2015 Option B+ guidelines recommend initiating lifelong ART for all pregnant and breastfeeding women, regardless of clinical or immunological status.^36^ Following its implementation, MTCT rates declined from 8.97% (95% confidence interval [CI]: 8.71–9.24) to 2.88% (95% CI: 5.03–9.34), reflecting a 25% relative reduction.^37^ PrEP has also emerged as an effective prevention of MTCT strategy for women at high risk of HIV acquisition during breastfeeding.^33^ A recent study in South Africa showed that a combined oral/long‐acting cabotegravir PrEP option was more effective than oral PrEP alone.^38^ Despite these advances, several challenges remain. A study from Guinea‐Bissau reported low awareness and limited knowledge of the prevention of MTCT among women, with only a minority demonstrating adequate understanding.^39^ In West and Central Africa, 68% of pediatric HIV infections were attributed to the absence of maternal ART, while in Eastern and Southern Africa, 32% of infections occurred due to maternal seroconversion during pregnancy or breastfeeding.^33^ Global differences in prevention of MTCT are presented in Table 3.^4^, ^13^, ^16^, ^38^ These regional disparities reflect strategic and systemic differences. In Eastern and Southern Africa, earlier adoption of Option B+, stronger integration of HIV services into maternal‐child health systems, and international support have led to broader ART coverage and improved outcomes.^37^ In contrast, West and Central Africa continue to face structural limitations, including weaker health infrastructure, lower antenatal care engagement, and inconsistent supply chains for ART.^33^ Fear of stigmatization and discrimination also remains a significant barrier to HIV testing, contributing to delayed diagnosis and treatment.^39^ Additionally, internalized stigma was shown to markedly reduce prevention of MTCT effectiveness (69% vs. 31%, *P* < 0.01).^40^ Promising developments are also underway in the field of HIV vaccine research. In macaque models, a CD4‐binding site immunogen capable of neutralizing broadly neutralizing antibody (bnAb) precursors marked an essential first step toward vaccine development.^41^ The HVTN 133 trial demonstrated that the membrane proximal external region bnAb lineage could be initiated after the second immunization, suggesting a viable path forward.^42^ Furthermore, the eOD‐GT8 60mer vaccine induced VRC01‐class bnAb precursors in nearly 97% of participants, providing clinical evidence for the effectiveness of germline‐targeting vaccine strategies.^43^ In Oxford, UK, a phase 1 trial of the HIVconsvX vaccine showed enhanced T cell proliferation and HIV‐1 inhibition upon antigen re‐exposure, signaling progress in T cell‐based vaccine design.^44^ Notably, in 2022, the first mRNA‐based HIV vaccine clinical trial was launched, marking a new frontier in vaccine technology.^45^ Continued investment in both prevention strategies and vaccine research is essential to sustain progress toward the elimination of pediatric HIV. Addressing persistent barriers, such as stigma, limited awareness, and gaps in access, will be critical to achieving equitable and effective prevention of MTCT worldwide

**TABLE 3:** Regional variations in PMTCT strategies

Region/Country	PMTCT strategy
World Health Organization (Global)	Option B+: Lifelong ART for all pregnant and breastfeeding women regardless of CD4 count or clinical stage
South Africa	Universal Option B+ with high ART coverage and integrated pre‐exposure prophylaxis access
China	Mandatory antenatal HIV screening; ART for HIV‐positive pregnant women and exposed infants
Uganda	Empirical ART for all HIV‐exposed infants, especially if mothers received ART during breastfeeding
West and Central Africa	PMTCT implementation varies. Coverage is often low due to systemic barriers.
Eastern & Southern Africa	High coverage of Option B+; integration with maternal‐child health systems

Abbreviations: ART, antiretroviral therapy; HIV, human immunodeficiency virus; PMTCT, prevention of mother‐to‐child transmission.

### Complications in pediatric HIV

ART in children, while life‐saving, is associated with several long‐term health complications, including dyslipidemia, glucose intolerance, and lipodystrophy.^46^ In a study conducted by Mukhuty et al., children aged 7–12 years who had been receiving a combination of two NRTIs with either PIs or NNRTIs for at least five years demonstrated significantly elevated systolic blood pressure, low‐density lipoprotein, total cholesterol, and body fat percentage compared to a control group (17% vs. 13.5%). Additionally, carotid intima‐media thickness, a marker of early atherosclerosis, was significantly greater in the ART group (0.481) than in controls (0.418), indicating increased cardiovascular risk.^47^ Current clinical guidelines for children receiving PIs, stavudine, or efavirenz recommend routine monitoring of fasting glucose and lipid profiles at baseline and 6 months after therapy initiation. Emphasis is also placed on encouraging regular physical activity to mitigate metabolic complications.^46^ In Uganda, Namuyonga et al.^48^ studied 285 HIV‐infected children aged 1–18 years on ART and found a range of cardiac abnormalities, including nonspecific T‐wave changes on electrocardiography and pericardial thickening, with or without effusion, on echocardiography. Further evaluation of cardiovascular risk in perinatally HIV‐infected children on ART has been explored through biomarker studies. Majonga et al.^49^ assessed inflammatory and cardiovascular biomarkers in children with perinatally acquired HIV and found that children with HIV exhibited higher levels of proinflammatory and cardiovascular markers compared to HIV‐uninfected peers, with increased C‐reactive protein and growth differentiation factor‐15 closely linked to the presence of cardiac abnormalities. ART may also affect renal disease due to the nephrotoxicity of the drugs (e.g., tenofovir), but renal problems may also result from HIV infection itself, as well as opportunistic infections.^50^ Renal complications have been observed in children receiving ART, with tenofovir being particularly associated with nephrotoxicity.^51^ However, several recent studies have reported no significant association between renal disease and either ART exposure or advanced stages of HIV infection,^52^, ^53^ suggesting the need for further research to clarify the relationship between ART and renal dysfunction. Neurologically, ART has reduced the incidence of HIV encephalopathy in children by 30%–50% compared to untreated individuals. Nonetheless, HIV‐infected children on ART still face an elevated risk of neurodevelopmental impairments, including language delays, lower intelligence quotient, reduced working memory, motor dysfunction, and visuomotor coordination deficits when compared to HIV‐negative peers.^54^

### Neurodevelopmental and cognitive impact of pediatric HIV

HIV infection in children is associated with significant neurodevelopmental and cognitive impairments, with outcomes strongly influenced by the timing and intensity of ART.^55^ Early and intensive ART has been identified as a key factor in mitigating these effects. According to Le Doaré et al., comprehensive reviews on the impact of HIV and ART exposure in children have demonstrated that those who received ART before 12 weeks of age exhibited cognitive outcomes comparable to the general population.^56^ However, subtle deficits in language expression, memory, and behavioral functioning persisted.^56^ On the other hand, Puthanakit et al., in a study involving 284 HIV‐infected children, found that those who had not received early ART showed poorer neurodevelopmental outcomes compared to HIV‐uninfected peers, reinforcing the importance of timely intervention.^55^ Similarly, untreated children demonstrated significantly worse motor and cognitive performance, scoring one to two standard deviations below the population mean, and faced an increased risk of developing HIV encephalopathy, highlighting the profound impact of delayed treatment.^56^ Additionally, in a study conducted by McHenry et al. in Kenya, children living with HIV showed significantly higher receptive language scores compared to HIV‐negative peers (*P* = 0.007).^57^ These findings suggest that targeted interventions may improve specific cognitive domains in HIV‐positive children, although disparities in overall neurodevelopment remain. Furthermore, the transition rates and neurocognitive outcomes in HIV‐infected children are presented in Table 4, comparing the impact of treatment on cognitive and motor outcomes, HIV encephalopathy risk, and transition success rates. Among this group of children, early diagnosis and prompt initiation of ART remain crucial to optimise neurocognitive outcomes in this population. Considerable further research is justified to refine these interventions and address current developmental challenges.

**TABLE 4:** Neurocognitive indicators and treatment outcomes in children with human immunodeficiency virus (HIV)

Indicator	Treated children	Untreated children
Cognitive scores	Close to the population mean	1–2 standard deviations below the population mean
Motor scores	Improved with antiretroviral therapy	Significantly lower
HIV encephalopathy risk	Lower	Higher
Transition success rate	Higher with structured programs	Lower without support

### Transitioning from pediatric to adult HIV care

The transition from pediatric to adult healthcare represents an important period for adolescents living with HIV, marked by complex psychological, social, and systemic challenges. One of the most pervasive barriers is HIV‐related stigma, which fosters fear of discrimination and rejection, often leading adolescents to conceal their status from both peers and healthcare providers. This fear can contribute to disengagement from adult care services, resulting in gaps in treatment adherence and clinical monitoring.^58^ Moreover, the psychological burden of managing a chronic illness is frequently compounded by depression, anxiety, and post‐traumatic stress disorder, which may be exacerbated during the transition to adult care.^59^ These mental health concerns, along with neurodevelopmental vulnerabilities associated with perinatal HIV infection, make it difficult for adolescents to meet the cognitive and emotional demands of navigating adult healthcare systems.^59^ Loss of long‐standing pediatric support networks during this period further intensifies these challenges. Adolescents often leave behind trusted healthcare providers and peer communities, leading to feelings of isolation and uncertainty in adult‐oriented care environments.^58^ Studies estimate that up to 60% of adolescents experience gaps in care during this transition, which can lead to poorer health outcomes and increased viral loads.^60^ A multidisciplinary, patient‐centered approach is essential to support adolescents during this transition. Integration of psychological support services, including counseling and peer support programs, can help address mental health concerns and promote resilience.^58^ Peer mentoring programs, which connect transitioning youth with individuals who have successfully navigated adult care, have shown particular promise in reducing isolation and improving engagement.^58^ Chew et al.^61^ reported that young people who received emotional and social support during the transition had better clinical outcomes and were significantly less likely to discontinue treatment. Specifically, those with strong support systems were 30% more likely to maintain viral suppression compared to unsupported peers. Population‐based data reinforces the importance of structured transition support. Ritchwood et al.^62^ reported that more than 70% of adolescents remained engaged in care one to two years post‐transition, while other studies reported retention rates below 55%. Interestingly, adolescents with special healthcare needs, females, and those with private insurance have higher odds of receiving transition services.^63^ The transition from pediatric to adult HIV care is an important phase with far‐reaching implications for the health and well‐being of adolescents living with HIV. A multidisciplinary approach, centered on psychological support, stigma reduction, and tailored healthcare services, is critical for ensuring that adolescents not only maintain engagement in care but also thrive into adulthood. The treatment‐related indicators for HIV, such as viral titer (measuring the eamount of HIV RNA in the blood), CD4+ count (assessing the number of CD4+ T‐cells, which play a key role in the immune system) and the impact of post‐traumatic stress disorder on treatment adherence and health outcomes, are presented in Table 5, highlighting the importance of systematic monitoring of effective ART. The transition from pediatric to adult HIV care is an essential phase with far‐reaching implications for the health and well‐being of adolescents living with HIV. A multidisciplinary approach, centered on psychological support, stigma reduction, and tailored healthcare services, is critical for ensuring that adolescents not only maintain engagement in care but also thrive into adulthood.

**TABLE 5:** The challenges and solutions for transitioning adolescents living with human immunodeficiency virus

Indicator	Description	Examples
**Medical**		
Retention in care	Dropout rates during transition	Retention rates drop below 55%
Viral load and CD4+ count	Indication of declining immune function	Declining CD4+ counts
Adherence to antiretroviral therapy	Challenges in maintaining ART adherence	Risk of drug resistance
**Psychological**		
Mental health issues	Increased prevalence of depression, anxiety, and post‐traumatic stress disorder	Heightened anxiety
Stigma and disclosure	Fear of stigma and its consequences	Non‐disclosure to providers
Loss of support networks	Feelings of isolation and anxiety	Leaving trusted providers
**Systemic**		
Healthcare system barriers	Insufficient adolescent‐specific training	Providers may not understand needs
Fragmented care	Challenges in navigating care	Separate services
**Solutions**		
Multidisciplinary approach	Incorporation of psychological support and peer programs	Support groups
Training for providers	Enhanced adolescent‐specific training	Better care
Gradual transition models	Facilitates a smoother transition through joint appointments and transitional clinics	Eases transition

### Conclusions and future directions

The management of pediatric HIV has undergone remarkable evolution over the past decades, driven by scientific advancements in virology, pharmacology, and public health policy. Despite these advances, important challenges persist. Long‐term ART in children is associated with metabolic, cardiovascular, renal, and neurodevelopmental complications, necessitating continuous monitoring and multidisciplinary management. The transition from pediatric to adult HIV care remains a particularly vulnerable period, often marked by loss of follow‐up, psychological stress, and treatment discontinuation. Future research should prioritize large‐scale, age‐stratified clinical trials to determine optimal dosing and safety of LAI therapies in infants and young children. Implementation studies are urgently needed to identify effective models for sustaining ART adherence in adolescents, especially during the transition to adult care. Further investigation into neurodevelopmental outcomes in children initiating ART in early infancy is essential, as are strategies to mitigate cognitive and behavioral sequelae. Research should also explore scalable interventions for improving prevention of MTCT coverage in high‐burden, resource‐limited regions, including the integration of maternal PrEP. Ongoing investment in vaccine research and immunoprophylaxis, along with community education and stigma reduction initiatives, will be key to achieving the ultimate goal of eliminating pediatric HIV.

## CONFLICT OF INTEREST

The authors declare no conflict of interest.
